# A convolutional neural network model of the neural responses of inferotemporal cortex to complex visual objects

**DOI:** 10.1186/1471-2202-12-S1-P35

**Published:** 2011-07-18

**Authors:** Satish Rohit, Srinivasa Chakravarthy

**Affiliations:** 1 Department of Biotechnology, Indian Institute of Technology, Madras, Chennai 600036, India

## 

We present a neural network model that replicates the response properties of the neurons in monkey inferior temporal cortex described in the studies of Tanaka and colleagues [1,2]. A convolutional neural network (CNN) known for its visual pattern recognition capabilities is used for this purpose. The present work consists of two studies.

In the first study, we simulate the “image reduction method” of [1] in order to study the responses of tuned neurons to complex visual patterns. The CNN used in this study consists of 4 hidden layers, 12 output neurons, and accepts a input image of size 50 X 50. The first hidden layer has 5 sub layers, each of size 46 X 46, and the third hidden layer has 12 sub layers, each of size 20 X 20. The network is trained on 12 images selected from the original study [1]. Neurons of the penultimate layer that exhibit a distinct response to one image, as opposed to all other images, are selected as *tuned neurons*. When reduced version of an image is presented, the corresponding tuned neurons preferentially show a drastic reduction in response; no such change is seen in the responses of a non-tuned neuron (fig. 1).

Next we investigate the inherent hierarchy of categorical representations of model neuron responses as in the experimental study of [2]. In this case, the CNN is trained on images of 12 categories used in [2], each consisting of about 15 sample images. The network consists of 6 hidden layers, 12 output neurons, and accepts an input image of size 50x50. The first hidden layer has 5 sub layers, each of size 46x46; the third hidden layer has 12 sub layers, each of size 20x20, and the fifth hidden layer has 12 hidden layers of size 6x6. We perform hierarchical clustering on the response vectors of neurons in the penultimate layer. Selected nodes in this tree are assigned one of the 12 low-level categories based on a score which is an average of two ratios (ratio1 = (number of category members under the node)/ (number of all members in the category) and ratio2 = (number of category members under the node)/ (number of all stimuli under the node)). The scores given to various categories in the model data bears a strong resemblance to the corresponding scores obtained in the experimental study.

**Figure 1:**
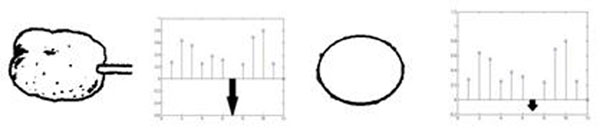
The highlighted arrow is the response of the tuned neuron to the image.

**Fig2:**
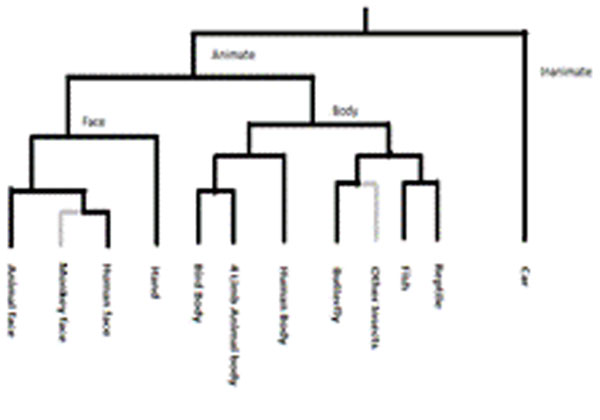
Hierarchical Tree

## References

[B1] Tanaka K Mechanisms of visual object recognition studied in monkeys Spatial Vision 2000 13 147 163 10.1163/156856800741171 11198228

[B2] Kiani R Esteky H Mirpour K Tanaka K Object Category Structure in Response Patterns of Neuronal Population in Monkey Inferior Temporal Cortex Journal of Neurophysiology 2007 97 4296 4309 10.1152/jn.00024.2007 17428910

